# Diagnostic Dilemma in Unusual Parasitic Infections and Their Presentations

**Published:** 2019

**Authors:** Tuhina BANERJEE

**Affiliations:** Department of Microbiology, Institute of Medical Sciences, Banaras Hindu University, Varanasi 221005, India

## Dear Editor-in-Chief

Parasitic diseases are a global health problem. “Warm countries are the worm countries” (1), the tropical climate of India makes it vulnerable for a wide diversity of human parasitic infections. The following report describes three situations in recent past where it was important to think beyond the usual parasitic infections and their presentations for a better understanding.

**Fig. 1:**
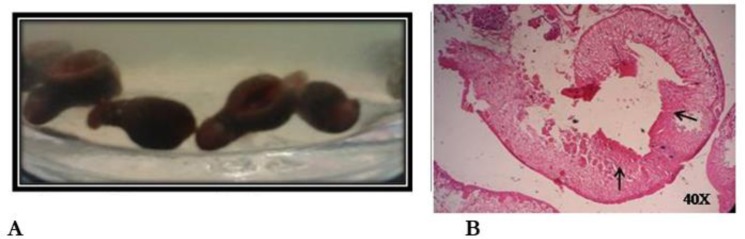
Adult worms and section of *G. hominis*. **A)** Adult worms of *G. hominis*; **B)** Cut section of *G. hominis* showing pharyngeal bulbs (arrows) and muscular pharynx

In the first case, a 20 yr male presented with passage of excess mucus in thick flaky forms in stool. Routine examination revealed the presence of mature proglottides and eggs of *Taenia* spp. in stool. He was advised for treatment with single dose of Praziquantel (400mg) and counseled on the probable modes of transmission of the disease. However, the patient denied any intake of meat and re-affirmed his strict vegetarian diet. His food was being cooked by a female cook for the last 3 months. Therefore, it was thought to be a case of symptomatic taeniasis due to *Taenia* infection in a strict vegetarian without any feature of neurological symptom or cysticercosis. In the second case, a 27 yr male who was being investigated for inflammatory bowel diseases presented with unidentified motile parasites with suckers in the caecum as an incidental finding in colonoscopy. Parasites were initially identified as trematodes with pyriform shaped body, conical anterior end, and oral sucker. On histological section, heart-shaped cross-section of the pharynx with pharyngeal bulbs and thick musculature was seen (Fig. 1). The parasite was confirmed as *Gastrodiscoides hominis*. However, repeated stool examination was negative. The patient admitted the habit of bathing in common pond water along with pigs. He has prescribed Praziquantel and was lost for follow up colonoscopy. The clinician could not ascertain the role of this parasite responsible for the symptoms in this case (2).

In the third case, a 4 months immunocompetent exclusively breastfed male infant presented with loose stool and mucous for 7 d and was investigated. Stool examination revealed the presence of cysts of *Giardia*. On interest of the parents, the likely source of *Giardia* was traced among the family members and environment of the affected child. Asymptomatic carriage of *Giardia* cysts was also seen in the mother of the infant. As the child was exclusively breastfed, the role of mother’s milk in the transmission of the parasites was suspected by the parents. However, further counseling and awareness of personal hygiene were done. These above cases represent the diagnostic dilemma in identification and justification of parasitic infections due to complex interplay between man and parasite life cycles. In the first case of taeniasis, the only explanation that could be thought of was in view of a very rare previously reported case of taeniasis with cysticercosis where tapeworm eggs have been transmitted from one human to other through poor hygiene (3).

In this case, cysticercosis could not be confirmed due to absence of symptoms and ethical issues involved in unwarranted investigations in asymptomatic patient. However, the wide prevalence of neurocysticercosis in India, where a significant population is vegetarian is supported by the fact of person to person transmission. Presently, plant-borne trematodiases are emerging worldwide due to abrupt changes in climate. In this context, gastrodiscoidiasis is extending its occurrence from Asia to Africa (4). This is acquired by ingestion of aquatic plants containing metacercaria stage of the parasite and there is no specific treatment. Though diagnosis is by coprology, cases are on record for no finding in stool which often makes the situation difficult for clinicians (5). The final case highlights two major issues. Firstly, the burden of the disease and potential reservoir in asymptomatic population and secondly the role of breast milk in protection against the infection. Anti-*Giardia* factors in breast milk prevent the attachment of trophozoites and establishment of infection in the intestinal epithelium of the infant. Further, in developing countries high titers of anti-*Giardia* secretory immunoglobulin (sIgA) in breast milk due to maternal exposure protect the infant from symptomatic infections thus helping in acquiring active immunity without overt presentations (6). However, in this case, high burden of parasites might have been responsible for the symptoms in the breastfed infants.

Evolution of parasites has been in parallel with evolution of man. Due to changes in natural and manmade environment along with climate, demography, behaviour, travel and importantly human encroachment into natural habitats of wildlife, the transmission dynamics, and ecology of these parasites are being affected and altered (1). Thorough knowledge of emerging parasites is needed.
